# The Mechanism of Starch Over-Accumulation in *Chlamydomonas reinhardtii* High-Starch Mutants Identified by Comparative Transcriptome Analysis

**DOI:** 10.3389/fmicb.2017.00858

**Published:** 2017-05-23

**Authors:** Kwang M. Koo, Sera Jung, Beom S. Lee, Jin-Baek Kim, Yeong D. Jo, Hong-Il Choi, Si-Yong Kang, Gook-H. Chung, Won-Joong Jeong, Joon-Woo Ahn

**Affiliations:** ^1^Advanced Radiation Technology Institute, Korea Atomic Energy Research InstituteJeongeup, South Korea; ^2^Department of Biological Sciences, Chonbuk National UniversityJeonju, South Korea; ^3^Molecular Biofarming Research Center, Korea Research Institute of Bioscience and BiotechnologyDaejeon, South Korea

**Keywords:** starch biosynthesis, glycolysis, comparative transcriptome analysis, microalgae, *Chlamydomonas reinhardtii*

## Abstract

The focus of this study was the mechanism of starch accumulation in *Chlamydomonas reinhardtii* high-starch mutants. Three *C*. *reinhardtii* mutants showing high-starch content were generated using gamma irradiation. When grown under nitrogen-deficient conditions, these mutants had more than twice as much starch than a wild-type control. The mechanism of starch over-accumulation in these mutants was studied with comparative transcriptome analysis. In all mutants, induction of *phosphoglucomutase 1* (*PGM1*) expression was detected; PGM1 catalyzes the inter-conversion of glucose 1-phosphate and glucose 6-phosphate in both starch biosynthetic and glycolytic pathway. Interestingly, transcript levels of *phosphoglucose isomerase 1* (*PGI1*), *fructose 1,6-bisphosphate aldolase 1* and *2* (*FBA1* and *FBA2*) were down-regulated in all mutants; PGI1, FBA1, and FBA2 act on downstream of glucose 6-phosphate conversion in glycolytic pathway. Therefore, down-regulations of *PGI1, FBA1*, and *FBA2* may lead to accumulation of upstream metabolites, notably glucose 6-phosphate, resulting in induction of *PGM1* expression through feed-forward regulation and that *PGM1* overexpression caused starch over-accumulation in mutants. These results suggest that PGI1, FBA1, FBA2, and PGM1 correlate with each other in terms of coordinated transcriptional regulation and play central roles for starch over-accumulation in *C*. *reinhardtii*.

## Introduction

The energy demands of the world are large and increasing and, for many reasons, cannot be met by traditional energy sources alone. Bioenergy is a promising alternative source of energy, particularly appealing as it is renewable. Biofuels are mostly obtained from food crops (Chisti, 2007; Sharma et al., 2012). However, because their production requires cultivable land, producers of bioenergy crops are in competition with food growers for agricultural lands. Microalgae have potential as a source for bioethanol and biodiesel (Chisti, 2007; de Faria Silva and Bertucco, 2016), and their growth does not require agricultural land (Li et al., 2008). These organisms produce a larger amount of biomass than do crop plants and green algae also produce starch, which can be used as the production of bioethanol (Doana et al., 2012).

Starch is a polysaccharide, a storage form of carbohydrate that is present in plastids, produced in the chloroplasts of higher plants and some algae during photosynthesis. Starch is composed of amylopectin and amylose. Amylopectin, a branched chain of glucose units, is the major component of starch. The other molecule that comprises starch is amylose, which can be helical or linear (Buleon et al., 1998). Starch biosynthesis has been widely studied in many plants, including the unicellular green alga *Chlamydomonas reinhardtii* (Buleon et al., 1998; Streb and Zeeman, 2012; Busi et al., 2015). To explore molecular mechanisms of starch and lipid biosynthesis in response to nitrogen deprivation in *C*. *reinhardtii*, transcriptomic and proteomic analysis were performed using starchless mutant *sta6*; this mutant displayed up-regulation of both glyoxylate and gluconeogenesis pathway under nitrogen starvation, which resulted in over-accumulation of TAG (Blaby et al., 2013; Goodenough et al., 2014; Schmollinger et al., 2014). In addition, systems biology analysis for *C*. *reinhardtii* in response to nitrogen starvation was reported by Park et al. (2015), which provided fundamental insight for starch and TAG biosynthesis. Schulz-Raffelt et al. (2016) reported *starch degradation 1* (*std1*) mutant showed hyper-accumulation of both starch and TAG under photoautotrophic condition under nutrient deprivation. *C*. *reinhardtii* produces starch by photosynthetic carbon fixation under phototrophic condition and also uses carbon-reduced compounds such as acetate under heterotrophic and mixotrophic condition (Johnson and Alric, 2013). There are two pathways for acetate assimilation to synthesis acetyl coenzyme A (Acetyl-CoA) in *C*. *reinhardtii*; acetyl-CoA synthetase (ACS) only involves in the one step conversion, or both acetate kinase (ACK) and phosphate acetyltransferase (PAT) participate in the two step conversion (Harris, 2009; Spalding, 2009; Johnson and Alric, 2013).

The glycolysis pathway, common to all cells, converts glucose to pyruvate. Several enzymes in the upper steps of the glycolysis pathway also participate in starch biosynthesis (Denis and Greyson, 1987). The glycolytic pathway is made up of three stages (Berg et al., 2002). In stage 1, glucose is converted to fructose 1,6-bisphosphate through three steps: phosphorylation, isomerization, and another phosphorylation. In stage 2, fructose 1,6-bisphosphate is cleaved into two three-carbon molecules. Finally, in stage 3, the three-carbon compounds are oxidized to pyruvate, generating ATP. In non-plants, glycolysis occurs in the cytoplasm; in plants, it takes place in both the cytoplasm and plastids. In higher plants, sucrose and starch are required as substrates for cytosolic and plastidic glycolysis, respectively (Plaxton, 1996). Some of the enzymes for cytosolic glycolysis do not appear to be present in many green algae species, which implies that sucrose is relatively less important compound for carbon flux in green algae (Singh et al., 2008).

In the present study, the molecular mechanism of starch over-accumulation was investigated using *C*. *reinhardtii* mutants displaying high-starch content through comparative transcriptome analysis. Key genes that regulate starch biosynthesis and glycolysis/gluconeogenesis for starch over-accumulation were identified. This comparative transcriptome study also offers fundamental information about starch biosynthesis and glycolysis/gluconeogenesis in green algae.

## Materials and Methods

### Mutant Generation and Culture Conditions

High-starch *C*. *reinhardtii* mutants (Sm142, Sm162, and Sm181) were used in this study, which were generated from *C*. *reinhardtii* strain cc124 (wild-type) by gamma irradiation. For generation of high-starch mutants, *C*. *reinhardtii* wild-type (strain cc124) cells grown in liquid Tris-acetate-phosphate (TAP) medium (Harris, 2009) were treated with 100 Gy gamma radiation for 1 h. The irradiated cells were spread onto solid TAP media and incubated for 10 days at 25°C under dim light. Over 500 colonies grown in TAP media were transferred onto solid nitrogen-deficient TAP (TAP-N; NH_4_Cl was replaced with KCl) media and incubated for 5 days to induce starch accumulation. To select high-starch mutant candidates, iodine vapor staining for colonies was performed. For selected mutant candidates, starch contents were analyzed using starch assay kit as mentioned below. For liquid culture used in this study, approximately 1 × 10^5^ cells were inoculated to 50 ml TAP medium. Culture was shaken continuously at 150 rpm at 25°C under constant white light (40 μmol m^-2^ s^-1^). For treatment of nitrogen starvation, 5-day-old sample grown in TAP medium was transferred to TAP-N medium and then cultured for 7 days under same condition as mentioned above. Siaut et al. (2011) reported that starch accumulation was rapidly increased for 2∼3 days and then gradually induced until day 7 after treatment of nitrogen deprivation. So, in this study, two time points (at days 2 and 7) were used to identify effect of nitrogen starvation on starch accumulation in *C*. *reinhardtii* mutants.

### Analysis of Transmission Electron Microscopy

Three-day-old cells grown in nitrogen-deficient TAP media were washed with 250 mM phosphate buffer (pH 7.0). Fixation was carried out using 1% (v/v) osmium tetroxide and 2.5% (v/v) glutaraldehyde. Graded ethanol series (50–100%) were utilized for dehydration. After dehydration, samples were embedded in Spurr’s resin and thin sections prepared using an ultramicrotome (Ultracut UCT, LEICA, Germany). Specimens were observed using a transmission electron microscope (TEM; Tecnai, FEI, Netherlands).

### Measurements of Growth Rate and Starch Content

Samples grown in nitrogen-deficient TAP media for 3 days were used. Starch content was evaluated using Starch assay kit (Sigma, St. Louis, MO, USA) according to manufacturer’s instruction. For measurement of growth rate, approximately 1 × 10^5^ cells were inoculated in 50 ml of TAP medium and incubated under the same condition as mentioned above. Optical density (OD) at 750 nm was measured every 24 h using a spectrophotometer (Shimazu, Kyoto, Japan).

### RNA Isolation

For RNA extraction, samples were taken at days 3 and 7 after transfer samples to nitrogen-deficient TAP media. Approximately 1 × 10^7^ cells were used for RNA extraction for each sample. RNA was extracted using TRIzol reagent (Invitrogen, Carlsbad, CA, USA) according to the manufacturer’s instructions. To remove genomic DNA contamination, one unit of RNase-free DNase (Takara, Kyoto, Japan) was treated to the total RNA for 30 min. RNA samples were purified according to the manufacturer’s instructions.

### RNA Sequencing and Data Analysis

Three biological replicates of each sample grown in nitrogen-deficient condition were prepared. Procedure of RNA isolation was followed as mentioned above. RNA-Seq paired end libraries were prepared using the Illumina TruSeq RNA Sample Preparation Kit v2 (Illumina, San Diego, CA, USA) according to the manufacturer’s instruction. The constructed library was quantified using the KAPA library quantification kit (Kapa Biosystems, Wilmington, MA, USA) following the manufacturer’s protocol. cDNA libraries were sequenced with Illumina *HiSeq2000* (San Diego, CA, USA). To generate unigene sequences, total RNA (10 μg) from each sample was prepared for creating a normalized cDNA and then amplified according to the Illumina RNA-seq protocol. Sequencing was carried out using Illumina HiSeq2000. Sequence data (quality of bp is upper than Q he) were selected using SolexaQA. All sequence reads from different samples were utilized to optimize the *de novo* assembly using the software Velvet (Zerbino and Birney, 2008) and Oases (Schulz et al., 2012). Transcripts of unigenes assembled with the total reads were validated by comparison with *C*. *reinhardtii* transcript sequences in Phytozome v11 (Plant Comparative Genomics portal of the Department of Energy’s [USA] Joint Genome Institute^1^) using BLASTx (*e*-value ≤ 1e^-10^). For short read mapping, reads were mapped to the assembled unigenes using the bowtie software (Langmead et al., 2009). The number of mapped clean reads for each assembled unigene was counted and then normalization of DESeq package performed (Anders and Huber, 2010). Identification of differentially expressed genes (DEGs) between a control and mutants was conducted using fold change and *t*-test data. False discovery rate (FDR) was applied to identify the threshold of the *p*-value in multiple tests using DESeq. Correlation and hierarchical clustering analysis were carried out via AMAP library in R. Functional enrichment analysis for Gene Ontology (GO) and KEGG pathway were conducted using Gene Ontology Database and DAVID^2^, respectively (Huang et al., 2009).

### Quantitative Reverse Transcription-PCR Analyses

Reverse transcription (RT) were performed using 5 μg total RNA with 200 units of superscript^®^III reverse transcriptase (Invitrogen, Carlsbad, CA, USA), according to manufacturer’s instruction. For quantitative RT-PCR, cDNA was amplified by SYBER Premix Ex Taq^TM^II (Takara, Kyoto, Japan) using CFX^TM^ Real-Time System (Bio-Rad, Hercules, CA, USA). Reactions were carried out with the following program: 40 cycles at 92°C for 20 s, at 55–60°C for 20 s and at 72°C for 20 s. To confirm maximum efficiency of PCR, standard curve analysis was conducted using serial dilutions of primer pairs. Primer sequences are as follows: *PGM1*, 5′-GAT TAC GAG GAG TGC GCC AG-3′ and 5′-GGG AGC CAT CTG TGA ACA CG-3′; *ACS2*, 5′-TGC TGC TGG GCT GTG CAT GA-3′ and 5′-ACG CAG ACA CGA GCG GCT CT-3′; *ACS3*, 5′-TGA TAA ATT GTC ACG TCG TT-3′ and 5′-GCC TAA ACG GGC CTA GTC-3′; *FBA1*, 5′-ATT TTG GGA GAG AGC GTT GAG-3′ and 5′-ACA ACA CCA GCA CAA AGC AC-3′; *FBA2*, 5′-CAG AGC AAC TGC AAC GAG AG-3′ and 5′-GCA CTT CAT CCC TGC TCT TC-3′; *STA1*, 5′-AAC GCC GAC ATC ACC ATC-3′ and 5′- TCT TGA ACA CGT AGA TGC CC-3′; *STA3*, 5′-TTC ATC GAG CCC AAG AAC G-3′ and 5′-AGT TGA GGT TGT GGA TGG TG-3′; *STA6*, 5′-CGT CTG TAT CCT CTG ACC AAG-3′ and 5′-TGG GTG AGG CAG TAA ATC TTG-3′; *TubA*, 5′-CTC GCT TCG CTT TGA CGG TG-3′ and 5′-CGT GGT ACG CCT TCT CGG C-3′. Specificity of PCR products was tested by melting curve analysis and gel electrophoresis. *TubA* was served as an internal control. Normalization and quantification were performed using Bio-Rad CFX manager 3.1 program (Bio-Rad, Hercules, CA, USA).

### Statistical Analyses

Statistical analyses for quantitative RT-PCR and growth measurement were performed with one-way analysis (ANOVA) using R program (version 3.25^3^).

## Results

### Identification for Starch Over-Accumulation in Mutants

**FIGURE 1:**
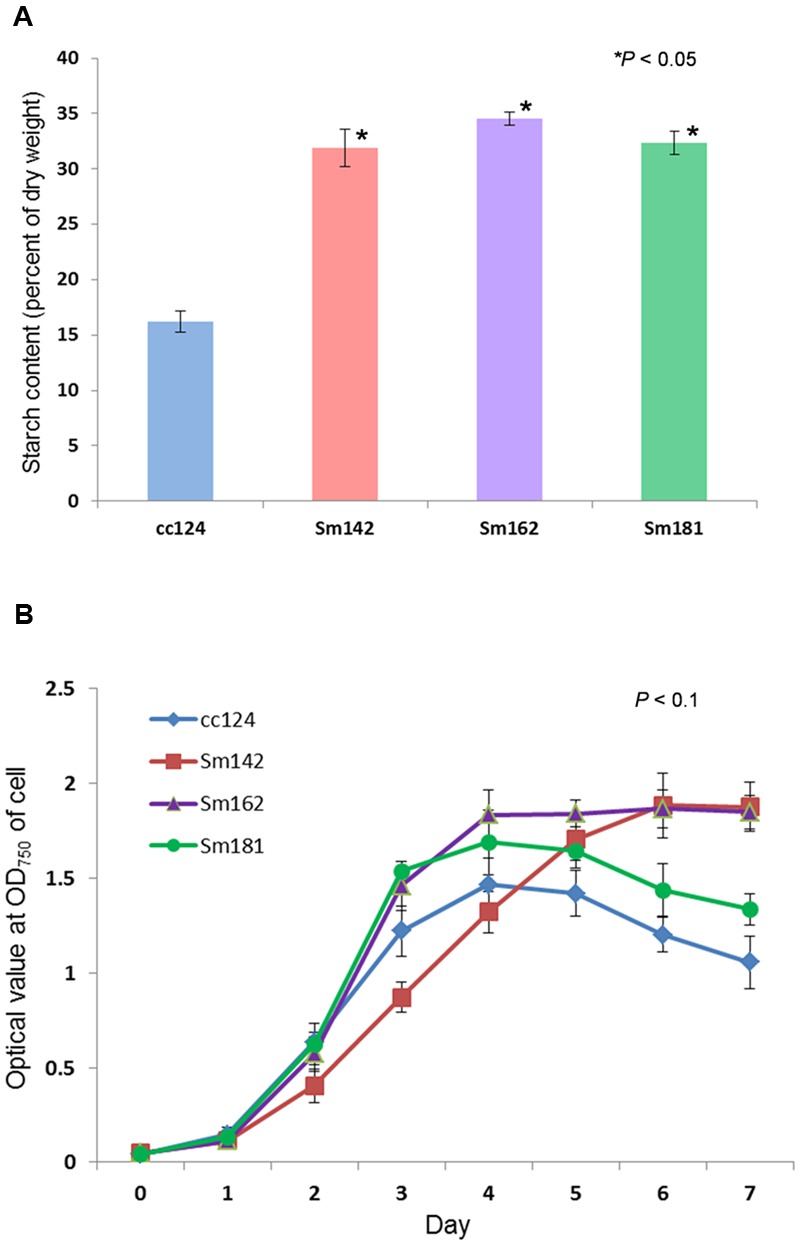
**Measurement of starch content and growth rate for mutants. (A)** Determination of starch content in high-starch mutants. Samples grown in nitrogen-deficient Tris-acetate-phosphate (TAP) media for 3 days were used. **(B)** Measurement of growth rate for high-starch mutants. Optical density (OD) values were measured at 750 nm using a spectrophotometer. **(A,B)** Values represent mean ± standard deviation (SD); *n* = 3. Wild-type control; cc124, high-starch mutants; Sm124, Sm162, and Sm181. Statistical analyses were conducted by one-way ANOVA.

**FIGURE 2:**
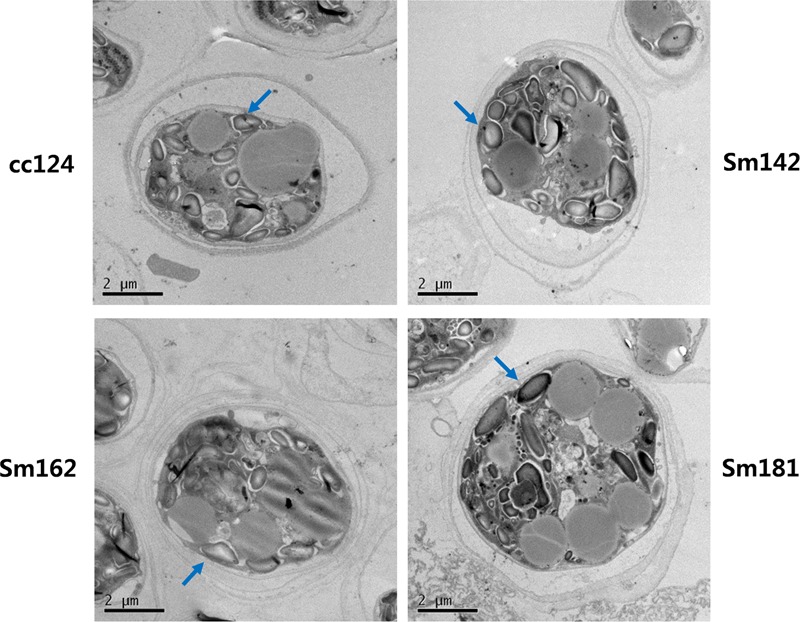
**Accumulation of starch granule in high-starch mutant cells.** Ultrastructure of mutant cells by TEM analysis. Arrows indicate starch granule. Mutants and wild-type cultured in nitrogen-deficient TAP media for 3 days were used. Wild-type control; cc124, high-starch mutants; Sm124, Sm162, and Sm181.

*Chlamydomonas reinhardtii* mutants showing high-starch contents were generated from *C*. *reinhardtii* strain cc124 (wild-type) after 100 Gy gamma irradiation (data not shown). Each mutant contained specific mutations compared to cc124 control, which was analyzed using transcriptome in/del analysis (Supplementary Table 1). Three mutants (Sm142, Sm162, and Sm181) displayed more than twice the amount of starch than cc124 wild-type control under nitrogen deprivation (**Figure 1A**). All mutants also showed higher starch contents than cc124 wild-type grown in nitrogen-replete condition (TAP media) for 5 days (data not shown). To determine growth rates of these mutants in TAP media, ODs were measured every 24 h for 7 days. Sm162 and Sm181 displayed similar growth rate with cc124 control until day 2, whereas Sm162 showed lower OD value than cc124 control until day 4 (**Figure 1B**). In the stationary phase, all mutants had greater OD values than a wild-type control. To identify morphological variation on starch accumulation in high-starch mutants, TEM analysis was also conducted (**Figure 2**). All mutants grown in nitrogen-deficient TAP media for 3 days accumulated large number of starch granules compared to cc124 control.

### RNA Sequencing and Comparison of Gene Expression between Mutants

**FIGURE 3:**
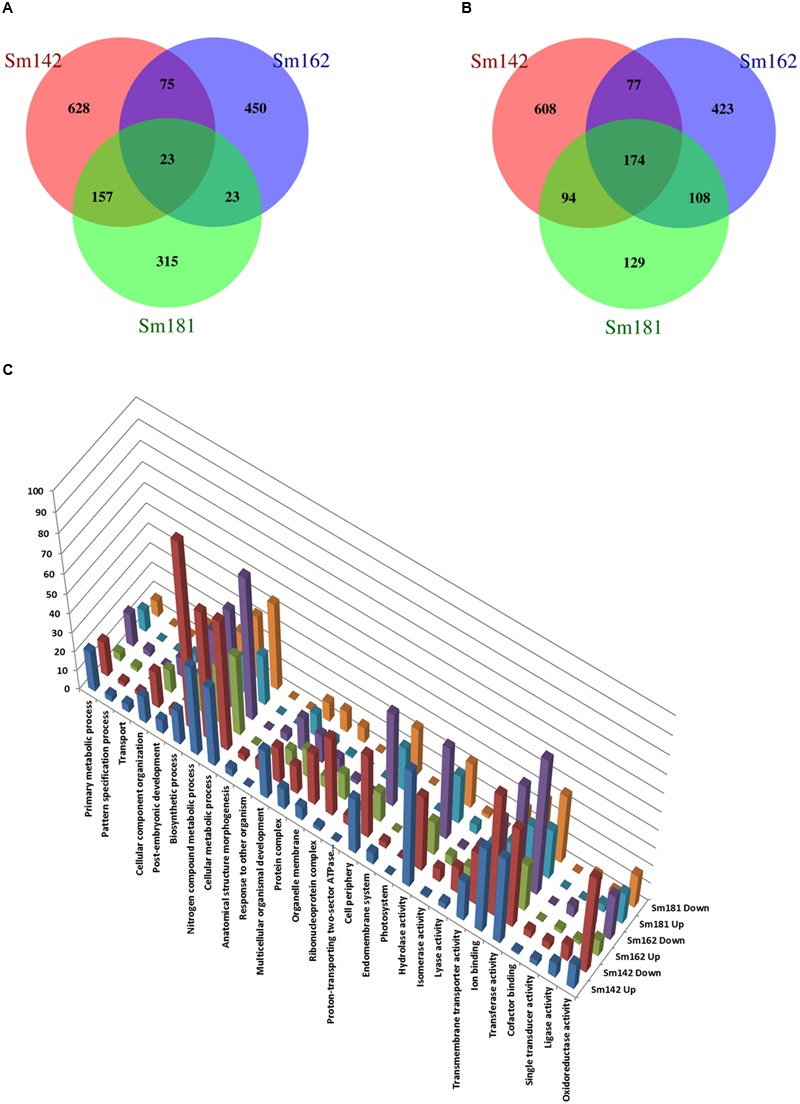
**Comparative differentially expressed genes (DEGs) analysis between high-starch mutants. (A–C)** Venn Diagrams display number of up- and down-regulated DEGs in mutants compared to cc124 control. Samples were grown in nitrogen-deficient TAP media for 3 days. **(A)** Number of up-regulated genes. **(B)** Number of down-regulated genes. **(C)** GO analysis of up- and down-regulated DEGs.

**FIGURE 4:**
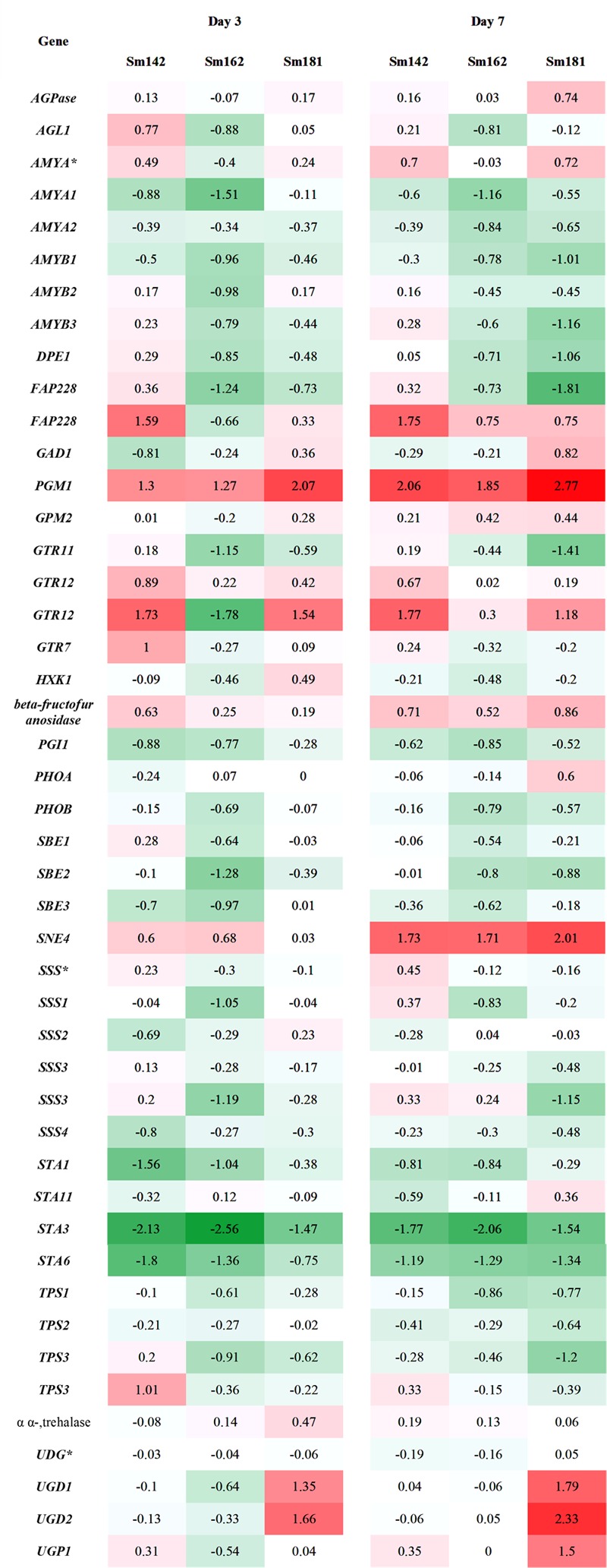
**Transcript levels of genes involved in starch biosynthesis in high-starch mutants by transcriptome analysis.** Samples incubated in nitrogen-deficient TAP media for 3 and 7 days. Values indicate fold changes (log_2_), which were calculated by comparison with each wild-type control grown in nitrogen-deficient TAP media. Red and green color indicate induction and reduction of gene expression, respectively. *AGPase, ADP-glucose pyrophosphorylase*; *AGL, alpha-glucosidase*; *AMY, alpha-amylase, DPE, 4-alpha-glucanotransferase*; *FAP228, flagellar associated protein*; *GAD, UDP-D-glucuronic acid decarboxylase*; *PGM, phosphoglucomutase*; *GTR, glycosyl transferase*; *HXK, hexokinase*; *PGI, phosphoglucose isomerase*; *PHO, starch phosphorylase*; *SBE, starch branching enzyme*; *SNE, NAD-dependent epimerase*/*dehydratase*; *SSS, soluble starch synthase*; *STA1, ADP-glucose pyrophosphorylase large subunit*; *STA11, 4-alpha-glucanotransferase*; *STA3, soluble starch synthase III*; *STA6, ADP-glucose pyrophosphorylase small subunit*; *TPS, trehalose-6-phosphate synthase/phosphatase*; *UGD, UDP-glucose dehydrogenase*; *UGP, UDP-glucose pyrophosphorylase*.

**FIGURE 5:**
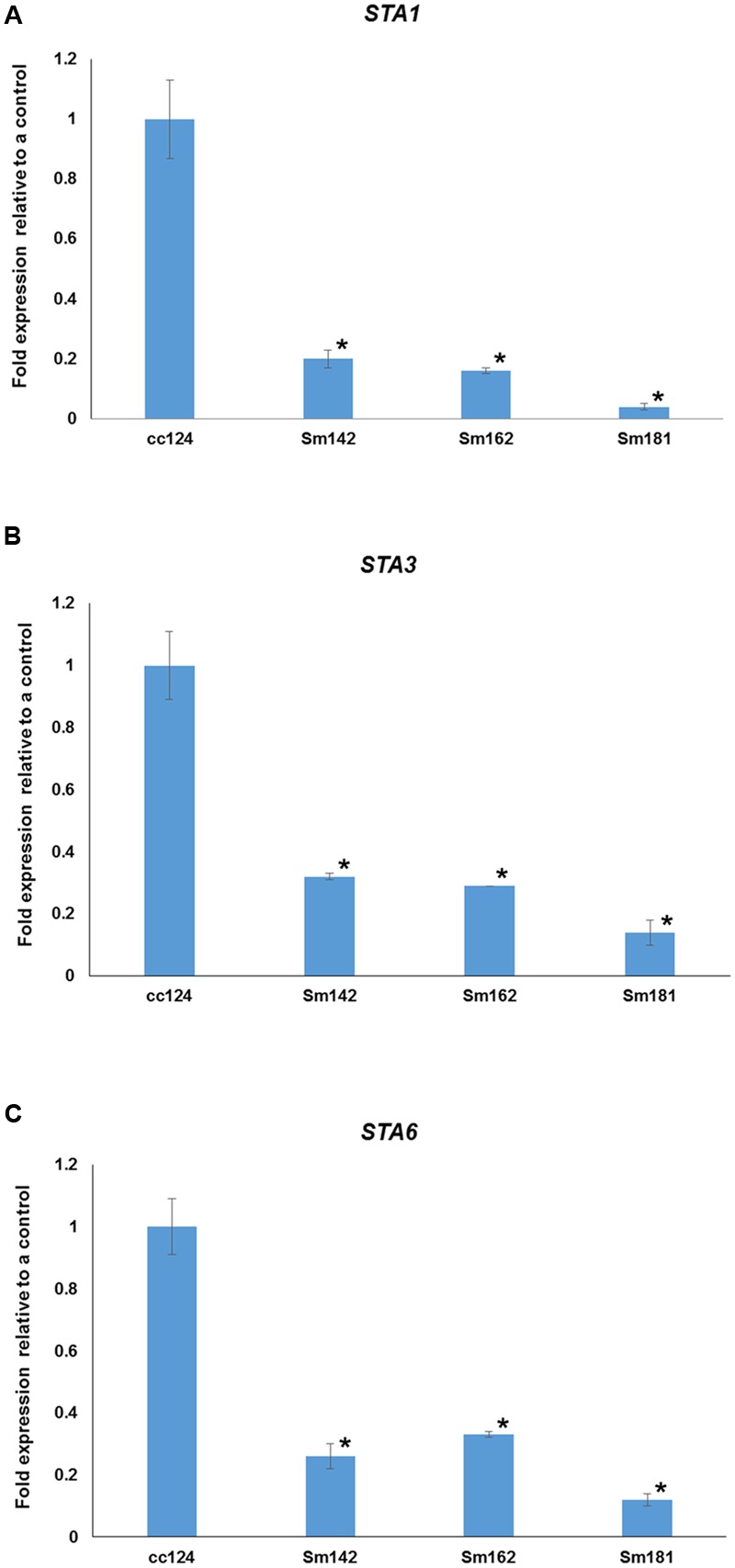
**Confirmation of starch synthase (STAs) expression in mutants. (A–C)** All mutants and cc124 control grown in nitrogen-deficient TAP media for 3 days were used. Transcript levels were determined by quantitative RT-PCR. Data are expressed as ± SD (*n* = 3). Statistical analyses were carried out by one-way ANOVA. ^∗^*p*-value < 0.05.

To investigate mechanism of starch over-accumulation in mutants, comparative transcriptome analysis was performed. From each sample, over 13 million trimmed reads were generated using RNA sequencing (**Table 1**), which provide at least 10× transcriptome coverage. Trimmed reads from each sample were mapped to the *C*. *reinhardtii* genome database (version 5.5) in Phytozome. All samples had over 95% mapped rates (**Table 1**). Three biological replicates were hired for reducing errors of sample preparation and sequencing. For comparison of gene expression between high-starch mutants and cc124 control, analysis of DEGs was performed (**Figure 3**). Sm142, Sm 162, and Sm 181 displayed 883, 571, and 518 up-regulated DEGs at day 3 after treatment of nitrogen starvation in comparison with cc124 control, respectively (**Figure 3A** and Supplementary Table 2). Twenty three up-regulated DEGs were commonly found in all mutants. Down-regulated DEGs at day 3 were also detected in Sm142 (953), Sm162 (782), and Sm181 (505); all mutants displayed 174 overlapped DEGs (**Figure 3B** and Supplementary Table 3). As shown in **Figures 4, 5**, total DEGs contained large number of genes encoding starch biosynthetic and glycolytic enzymes. Treatment of nitrogen starvation for 7 days reduced total number of up- and down-regulated DEGs compared to these from day 3, however, overlapped DEGs in mutants increased (data not shown). Up- and down-regulated DEGs from samples at day 3 were used for GO enrichment analysis using GO Database. Large numbers of DEGs at day 3 were classified as nitrogen compound metabolic process, cellular metabolic process, and transferase activity of GO Terms (**Figure 3C** and Supplementary Table 4).

**Table 1:** Total number of trimmed and mapped reads for control and each mutant grown in nitrogen-deficient condition.

Sample	Total trimmed reads^∗^	Mapped read	Mapped rate (%)
cc124	20,527,427	19,813,851	96.5
sm142	18,584,829	17,785,911	95.7
sm162	19,517,890	18,748,577	96.1
sm181	13,033,402	12,554,252	96.3

*^∗^All trimmed read were summed from the three biological replicates of each sample.*

### Expression Pattern of Genes Participated in Starch Biosynthesis and Glycolysis/Gluconeogenesis in Mutants

To understand mechanism of starch over-accumulation in mutants, expression patterns of genes on starch biosynthesis were investigated by KEGG pathway mapping using transcriptome data (**Figure 4** and Supplementary Table 5). High induction of *phosphoglucomutase 1* (*PGM1*) encoding starch biosynthetic enzyme was found in all mutants at days 3 and 7 after treatment of nitrogen starvation (**Figure 4**). Whereas, transcript level of *phosphoglucose isomerase 1* (*PGI1*) also decreased in all mutants at days 3 and 7. Surprisingly, key genes encoding starch biosynthetic enzymes, such as *ADP-glucose pyrophosphorylase large subunit* (*STA1*), *soluble starch synthase III* (*STA3*), and *ADP-glucose pyrophosphorylase small subunit* (*STA6*), were significantly down-regulated in all mutants (**Figure 4**). Quantitative RT-PCR analysis was performed to confirm expression levels of these genes. All mutants displayed down-regulations of *STA1, STA3*, and *STA6* expression at day 3 after treatment of nitrogen starvation (**Figure 5**). **Figure 4** also displays reductions of *alpha-* and *beta-amylase* (*AMYA1, AMYA2*, and *AMYB1*) transcript in mutant at days 3 and 7.

**FIGURE 6:**
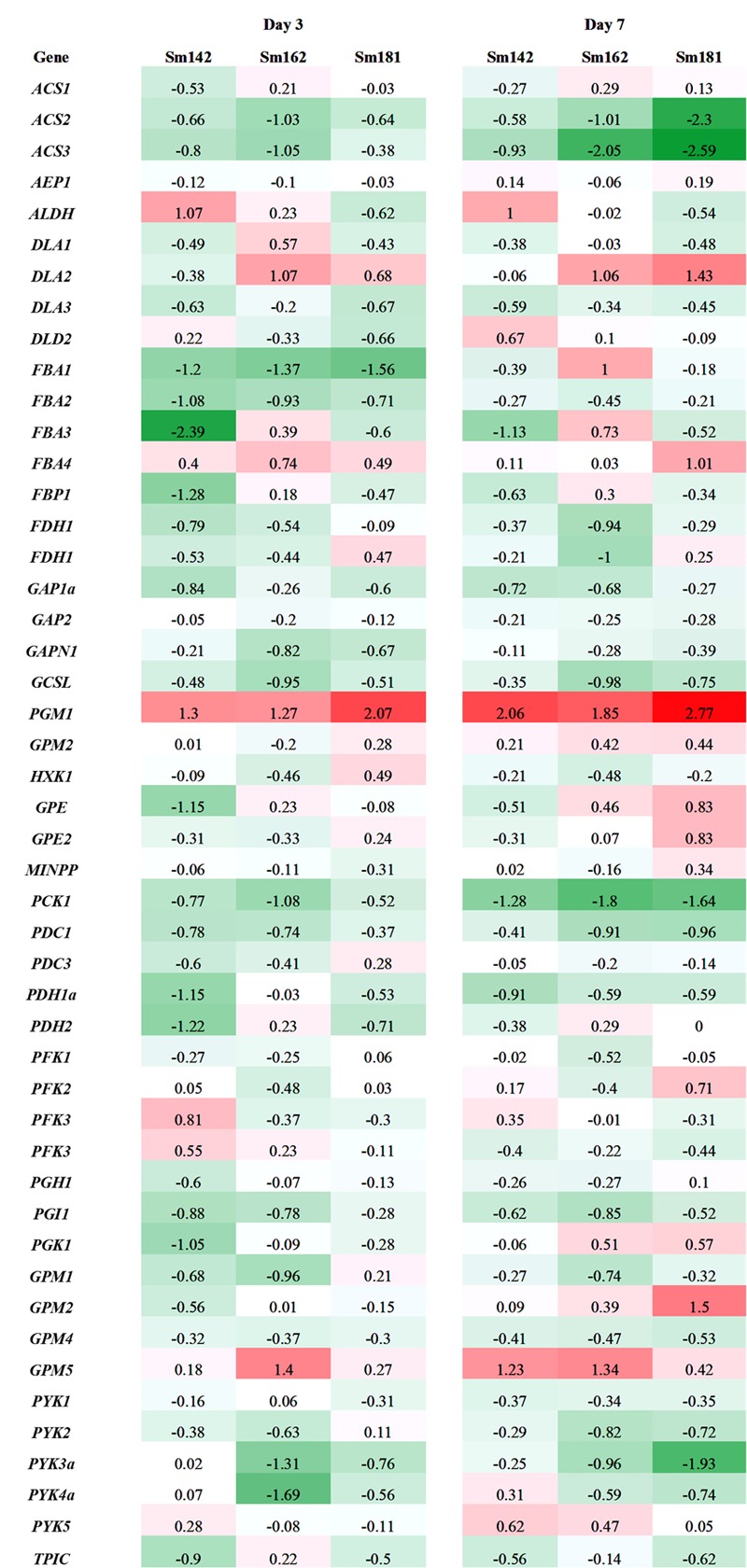
**Expression levels of genes participated in glycolysis/gluconeogenesis in high-starch mutants by transcriptome analysis.** Mutants and wild-type control were incubated in nitrogen-deficient TAP media for 3 and 7 days. Values indicate fold changes (log_2_), which were calculated by comparison with each wild-type control. Red and green color indicate induction and reduction of gene expression, respectively. *ACS, acetyl-CoA synthetase*; *AEP, aldose-1-epimerase*; *ALDH, aldehyde dehydrogenase*; *DLA, dihydrolipoamide acetyltransferase*; *FBA, fructose-1,6-bisphosphate aldolase*; *FBP, fructose-1,6-bisphosphatase*; *FDH, formaldehyde dehydrogenase*; *GAP, glyceraldehyde 3-phosphate dehydrogenase*; *GAPN, glyceraldehyde 3-phosphate dehydrogenase non-phosphorylating*; *GCSL, dihydrolipoyl dehydrogenase*; *PGM, phosphoglucomutase*; *HXK, hexokinase*; *GPE*; *glucose-6-phosphate 1-epimerase*; *MINPP, multiple inositol-polyphosphate phosphatase*; *PCK, phosphoenolpyruvate carboxykinase*; *PDC, mitochondrial pyruvate dehydrogenase complex*; *PDC, mitochondrial pyruvate dehydrogenase complex E1 component alpha subunit*; *PDH, pyruvate dehydrogenase E1 beta subunit*; *PFK, phosphofructokinase family protein*; *PGH, enolase*; *PGI*; *phosphoglucose isomerase*; *PGK, phosphoglycerate kinase*; *GPM, phosphoglycerate mutase*; *PYK, pyruvate kinase*; *TPIC, triose phosphate isomerase*.

**FIGURE 7:**
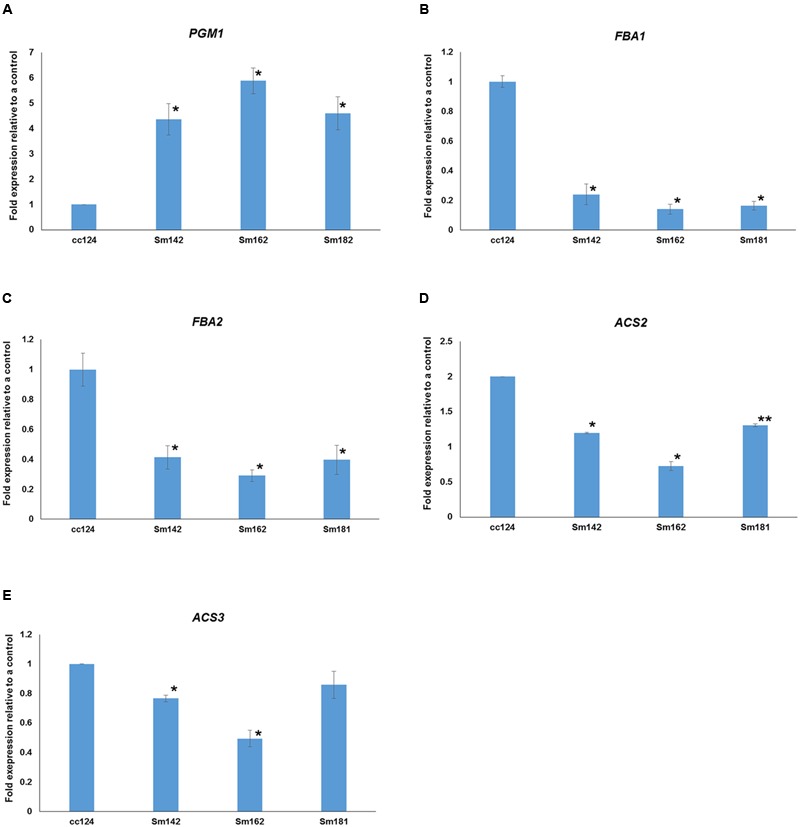
**Expressional confirmations for key genes involved in starch over-accumulation in mutants. (A–E)** All mutants and cc124 control grown in nitrogen-deficient TAP media for 3 days were used. Transcript levels were determined by quantitative RT-PCR. Data are expressed as ± SD (*n* = 3). Statistical analyses were conducted by one-way ANOVA. ^∗^*p*-value < 0.05, ^∗∗^*p*-value < 0.1.

Expression levels of 48 genes which participate in glycolytic/gluconeogenic pathway including post-glycolytic process were determined between mutants by heatmap analysis (**Figure 6** and Supplementary Table 6). *Fructose 1,6-bisphosphate aldolase 1* and *2* (*FBA1* and *FBA2*) expression were significantly reduced at day 3 in all mutants in comparison with cc124 control (**Figure 6**). *FBA2* transcript was also down-regulated at day 7, whereas no consistent pattern of *FBA1* expression was observed between mutants. Expression levels of *phosphoenolpyruvate carboxykinase 1* (*PCK1*), *mitochondrial pyruvate dehydrogenase complex 1* (*PDC1*), and *acetyl-CoA synthetases 1* and *2* (*ACS2* and *ACS3*) were down-regulated at days 3 and 7 in all mutants (**Figure 6**). To confirm expression levels of these genes, quantitative RT-PCR analysis was carried out. **Figure 7** shows expression patterns of *PGM1, FBA1, FBA2, ACS2*, and *ACS3* at day 3 after treatment of nitrogen starvation in each mutant. *PGM1* expression were highly up-regulated over fourfold in each mutant compared to a wild-type control. Transcript levels of *FBA1, FBA2*, and *ACS2* were significantly reduced. Down-regulations of *ACS3* transcripts were found in Sm162 mutant, but Sm142 and Sm181 showed slight reduction of *ACS3* expression. Expression patterns of these genes confirmed by quantitative RT-PCR analysis were similar with these by the transcriptome analysis.

## Discussion

### PGM1 Plays a Key Role in Starch Accumulation in *C*. *reinhardtii*

To investigate starch over-accumulation mechanism in high-starch mutants, 46 genes involved in starch biosynthetic pathway were selected from the RNA sequencing data by KEGG pathway mapping. Expression levels of these genes between mutants are shown in **Figure 4**. We observed a large amount of the transcript of *PGM1* in all three mutants compared to a wild-type control under nitrogen-deficient condition (**Figures 4, 6**). PGM1 catalyzes the inter-conversion between glucose 1-phosphate and glucose 6-phosphate in starch biosynthetic pathway (Periappuram et al., 2000). Plastidial PGM1 appears to be an important enzyme in starch biosynthesis in higher plants as well as in *C. reinhardtii*. Overexpression of *Arabidopsis* plastidial *PGM* enhanced starch accumulation in tobacco (Uematsu et al., 2012) and the *PGM1* knock-out mutant had lower starch content than a wild-type plant in *Arabidopsis* (Streb et al., 2009). Park et al. (2015) also observed that, at the initial stages of nitrogen deprivation, the *PGM1* transcript was up-regulated compared to the control that was not under nitrogen deprivation in *C. reinhardtii*. These studies indicate that PGM1 is important in starch accumulation in both higher plants and green algae. Therefore, we suggest that up-regulation of *PGM1* enhanced conversion of glucose 6-phosphate to glucose 1-phosphate, which resulted in starch over-accumulation in all mutants.

We were surprised that the transcript levels of other known genes essential for starch biosynthesis, such as *STA3* and *STA6*, were reduced at days 3 and 7 after treatment of nitrogen deprivation in all mutants (**Figures 4, 5**). STA6 catalyzes the conversion of ATP and glucose 1-phosphate to ADP-glucose (Espada, 1962; Zabawinski et al., 2001), and STA3 mediates amylose synthesis from ADP-glucose (Fontaine et al., 1993). The activities of soluble starch synthases were in inverse proportion to starch content in higher plant (Park and Nishikawa, 2012). In *C*. *reinhardtii*, reduced expression of both *soluble starch synthase* and *ADP-glucose pyrophosphorylase* were also detected by increasing the time of nitrogen starvation (Park et al., 2015). Thus, it may be that expression of *STA3* and *STA6* depend on starch content. If so, down-regulation of *STA3* and *STA6* in mutants resulted from feedback regulation by the accumulated starch content.

### Suppression of Glycolytic Pathway Enhances Starch Accumulation

Down-regulations of amylase *alpha-* and *beta-amylase* (*AMYA1, AMYA2*, and *AMYB1*) transcripts were observed in mutant at days 3 and 7 (**Figure 4**). Alpha-amylase is a key enzyme that involves in degradation of storage starch granule during seed germination in higher plants (Lloyd et al., 2005). Beta-amylase regulates breakdown of transient starch granule in leaves. In leaves of *Arabidopsis* and potato, starch over-accumulation was observed by the absence of beta-amylase, due to slow starch degradation at the dark cycle (Scheidig et al., 2002; Fulton et al., 2008). Furthermore, hyper-accumulations of both starch and TAG were found in *C*. *reinhardtii std1* mutant (Schulz-Raffelt et al., 2016). Therefore, reductions of *alpha-* and *beta-amylase* may affect to starch over-accumulation in mutants. Expression patterns of genes involved in glycolysis/gluconeogenesis were evaluated between mutants by heatmap analysis (**Figure 6**), because glycolytic pathway is working in conjunction with starch breakdown. Starch biosynthetic and glycolytic reaction are catalyzed by some of the same enzymes (Denis and Greyson, 1987). PGI1 are enzymes that participate in both glycolytic/gluconeogenic and starch biosynthetic pathway. PGI catalyzes the inter-conversion of glucose-6 phosphate and fructose-6 phosphate, which mediates connection of Calvin cycle to both starch biosynthetic pathway (Dietz, 1985). *Arabidopsis* mutant *pgi1* showed low starch content in leave (Bahaji et al., 2015), which implies critical role of *PGI1* in starch biosynthesis. Interestingly, in this study, reduced expression of *PGI1* was determined in high-starch mutants by transcriptome and quantitative RT-PCR analysis (**Figures 6, 7**). Possible explanation of starch over-accumulation in mutants is that the down-regulation of PGI1 may reduce conversion of glucose-6 phosphate to fructose-6 phosphate in mutant cells, which could cause accumulation of glucose 6-phosphate. Accumulation of glucose 6-phosphate may have caused the up-regulation of *PGM1* that we observed (**Figures 4, 6, 7**), through feed-forward regulation. This, in turn, could lead to starch over-accumulation in mutants.

Under nitrogen deprivation, *FBA1* and *FBA2* expression were determined at day 3 in all mutants compared to a wild-type control (**Figures 6, 7**). Furthermore, *FBA2* transcript was slightly down-regulated at day 7. FBA catalyzes conversion of fructose 1,6-bisphosphate to glyceraldehyde 3-phosphate and dihydroxyacetone phosphate in glycolysis (Rutter, 1964). An interesting point to note here is that Periappuram et al. (2000) reported the inhibition of PGM enzyme activity by fructose 1,6-bisphosphate and RuBP through *in vitro* enzyme assay, which implicates that fructose 1,6-bisphosphate may act as intermediate compound for Calvin cycle and starch biosynthetic pathway, especially control of PGM1 activity. This previous study supports our result that there is the correlation between *PGM1, FBA1*, and *FBA2* expression in glycolytic and starch biosynthetic pathway.

We did not detect any consistent patterns in the expression of *FBA3* and *FBA4* between mutants compared to expression of *FBA1* and *FBA2* (**Figure 6**). In *Arabidopsis*, the *FBA* gene family is classified into plastidic and cytosolic type and expression patterns of these genes varied with developmental stages and stresses (Lu et al., 2012). Recently, Park et al. (2015) analyzed transcriptome to nitrogen deprivation in *C*. *reinhardtii* and noted differential expression of *FBA* genes in response to time of nitrogen deprivation. The differential expression of *FBA* genes implicates functional diversity of FBA family in *C*. *reinhardtii*. Several gene transcripts encoding glycolytic/gluconeogenic enzymes (including post-glycolytic process) were significantly down-regulated, such as *PCK1, ACS2*, and *ACS3* (**Figures 6, 7**). Gluconeogenic enzyme PCK1 catalyzes oxaloacetate to phosphoenolpyruvate and ACS converts acetate to acetyl-CoA in acetate metabolism (Blaby et al., 2013). Similarly with our observation, Blaby et al. (2013) also reported inductions of *PCK1* and *ACS3* transcript in *C*. *reinhardtii* starchless mutant *sta6* under nitrogen-deficient condition. Thus, the reason of reductions of *PCK1, ACS2*, and *ACS3* transcript in high-starch mutants may be due to inverse expression against starch content.

## Conclusion

**FIGURE 8:**
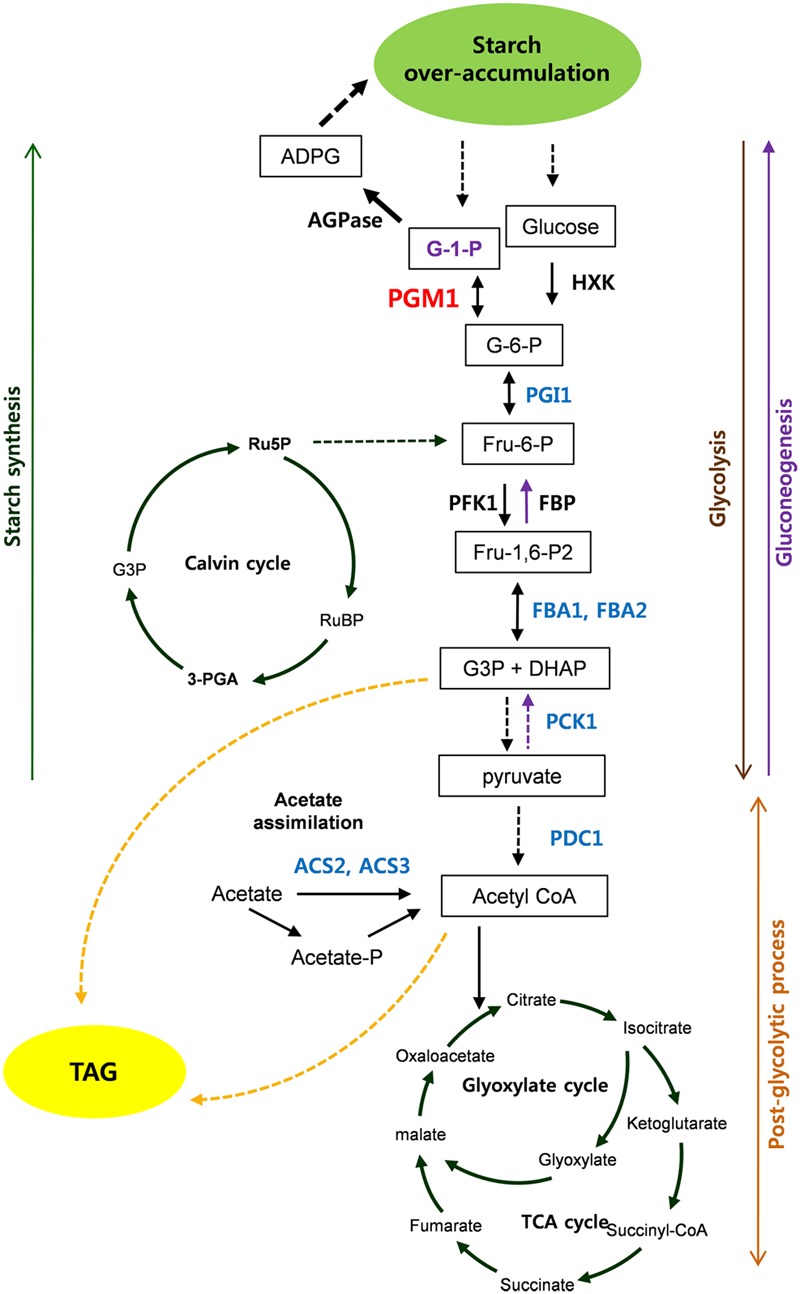
**Scheme of starch over-accumulation in *Chlamydomonas reinhardtii* high-starch mutants.** Red and blue color indicate induction and reduction of gene expression, respectively.

Based on our results, we propose the scheme of starch over-accumulation in *C*. *reinhardtii* mutants in **Figure 8**. In short, aberrant expression of *PGM1, PGI1, FBA1*, and *FBA2*, encoding starch biosynthetic and glycolytic enzymes, were detected in high-starch mutants. Reductions of *PGI1, FBA1*, and *FBA2* expression caused down-regulation of glycolytic pathway, which may consequently result in accumulation of glucose 6-phosphate. Induction of *PGM1* expression is likely due to feed-forward regulation by high level of glucose 6-phosphate content, which enhances starch over-accumulation. Therefore, we propose that PGM1, PGI1, FBA1, and FBA2 correlate each other and play key roles in starch accumulation in *C*. *reinhardtii*. Furthermore, PCK1, ACS2, and ACS3 seem to affect starch over-accumulation. This study provides fundamental information of glycolysis/gluconeogenesis and starch biosynthesis in green algae.

## Author Contributions

J-WA and W-JJ designed experiments and wrote the manuscript. KK, SJ, and BL performed experiments. J-BK, YJ, H-IC, S-YK, and G-HC commented on the manuscript and analyzed data. All authors contributed to writing the manuscript.

## Conflict of Interest Statement

The authors declare that the research was conducted in the absence of any commercial or financial relationships that could be construed as a potential conflict of interest.

## Footnotes

1 http://www.phytozome.net/

2 http://david.abcc.ncifcrf.gov/tools.jsp

3 http://www.r-project.org/
