# Supplementary material for: The Mechanism of Starch Over-Accumulation in Chlamydomonas reinhardtii High-Starch Mutants Identified by Comparative Transcriptome Analysis
Source: Front Microbiol. 2017 May 23;8:858. doi: 10.3389/fmicb.2017.00858 (PMC5440458; doi:10.3389/fmicb.2017.00858)
Supplement: Supplementary file 1 [file Table_1.DOCX]

| **Supplemental Table 1.** In/del analysis to identify different mutations in each mutant. | | | | | | | | | |  |  |
| --- | --- | --- | --- | --- | --- | --- | --- | --- | --- | --- | --- |
|  | |  |  |  |  |  |  |  |  |  |  |
| **Chromosome** | **Position** | | **Sm181** | **Sm181_Depth** | **Sm162** | **Sm162_Depth** | **Sm142** | **Sm142_Depth** | **CC124** | **CC124_Depth** | **Genic/Intergenic** |
| Cre11.g481900.t1.2 | 1230 | | * | 0\|20 | * | 0\|16 | +5CGGGA/* | 12\|22 | * | 0\|33 | Cre11.g481900.t1.2 |
| Cre06.g259500.t1.1 | 10621 | | -6CACACG | 3\|3 | * | 0\|1 | - | 0 | * | 0\|2 | Cre06.g259500.t1.1 |
| Cre17.g706450.t1.1 | 2474 | | * | 0\|4 | +3CAG/* | 4\|6 | */+3CAG | 3\|8 | */+3CAG | 4\|9 | Cre17.g706450.t1.1 |
| Cre03.g174050.t1.2 | 4148 | | * | 0\|21 | * | 0\|25 | -5CCATC | 16\|16 | * | 0\|39 | Cre03.g174050.t1.2 |
| Cre03.g178276.t1.2 | 527 | | * | 0\|31 | * | 0\|24 | -7CCACCTT/* | 43\|46 | * | 0\|31 | Cre03.g178276.t1.2 |
| Cre01.g023800.t1.1 | 7041 | | * | 0\|10 | -12CTGGACGCCGAC | 5\|5 | * | 0\|8 | * | 0\|32 | Cre01.g023800.t1.1 |
| Cre01.g042450.t1.1 | 8569 | | * | 0\|24 | -1G/* | 31\|33 | * | 0\|38 | * | 0\|29 | Cre01.g042450.t1.1 |
| Cre10.g454000.t1.2 | 8873 | | * | 0\|2 | * | 0\|6 | -1G | 3\|3 | * | 0\|6 | Cre10.g454000.t1.2 |
| Cre12.g495956.t1.1 | 2735 | | * | 0\|1 | -1G | 3\|3 | -1G | 5\|5 | -1G | 5\|5 | Cre12.g495956.t1.1 |
|  | |  |  |  |  |  |  |  |  |  |  |
