# Supplementary material for: The Mechanism of Starch Over-Accumulation in Chlamydomonas reinhardtii High-Starch Mutants Identified by Comparative Transcriptome Analysis
Source: Front Microbiol. 2017 May 23;8:858. doi: 10.3389/fmicb.2017.00858 (PMC5440458; doi:10.3389/fmicb.2017.00858)
Supplement: Supplementary file 4 [file Table_4.DOCX]

**Supplemental Table 4.** GO analysis of differentially expressed genes in three starch mutants after treatment of nitrogen starvation for 3 days.

| **Category^*^** | **GO ID** | **GO Term** | **Sm142** | | **Sm162** | | **Sm181** | |
| --- | --- | --- | --- | --- | --- | --- | --- | --- |
|  |  |  | **Up** | **Down** | **Up** | **Down** | **Up** | **Down** |
| BP | GO:0044238 | primary metabolic process | 21 | 18 | 4 | 17 | 11 | 8 |
| BP | GO:0007389 | pattern specification process | 3 | 2 | 2 | 3 | 0 | 0 |
| BP | GO:0006810 | transport | 4 | 2 | 0 | 0 | 0 | 0 |
| BP | GO:0016043 | cellular component organization | 14 | 19 | 12 | 10 | 4 | 12 |
| BP | GO:0009791 | post-embryonic development | 7 | 4 | 0 | 6 | 1 | 4 |
| BP | GO:0009058 | biosynthetic process | 18 | 96 | 20 | 36 | 17 | 19 |
| BP | GO:0006807 | nitrogen compound metabolic process | 47 | 67 | 31 | 52 | 20 | 33 |
| BP | GO:0044237 | cellular metabolic process | 42 | 68 | 42 | 74 | 26 | 45 |
| BP | GO:0009653 | anatomical structure morphogenesis | 4 | 3 | 0 | 0 | 0 | 0 |
| BP | GO:0051707 | response to other organism | 0 | 6 | 1 | 4 | 0 | 0 |
| BP | GO:0007275 | multicellular organismal development | 26 | 19 | 9 | 18 | 12 | 10 |
| CC | GO:0043234 | protein complex | 11 | 15 | 15 | 15 | 4 | 11 |
| CC | GO:0031090 | organelle membrane | 7 | 28 | 6 | 8 | 1 | 8 |
| CC | GO:0030529 | ribonucleoprotein complex | 2 | 42 | 14 | 2 | 0 | 0 |
| CC | GO:0033178 | proton-transporting two-sector ATPase complex | 0 | 5 | 0 | 0 | 0 | 0 |
| CC | GO:0071944 | cell periphery | 30 | 45 | 16 | 50 | 23 | 25 |
| CC | GO:0012505 | endomembrane system | 6 | 3 | 0 | 0 | 0 | 0 |
| CC | GO:0009521 | photosystem | 0 | 0 | 10 | 0 | 0 | 0 |
| MF | GO:0016787 | hydrolase activity | 64 | 41 | 18 | 50 | 26 | 24 |
| MF | GO:0016853 | isomerase activity | 1 | 7 | 5 | 1 | 5 | 2 |
| MF | GO:0016829 | lyase activity | 3 | 14 | 5 | 8 | 0 | 0 |
| MF | GO:0022857 | transmembrane transporter activity | 22 | 29 | 12 | 30 | 7 | 17 |
| MF | GO:0043167 | ion binding | 47 | 67 | 22 | 54 | 23 | 28 |
| MF | GO:0016740 | transferase activity | 48 | 55 | 26 | 74 | 27 | 37 |
| MF | GO:0048037 | cofactor binding | 1 | 4 | 0 | 0 | 0 | 0 |
| MF | GO:0004871 | single transducer activity | 3 | 4 | 2 | 6 | 0 | 5 |
| MF | GO:0016874 | ligase activity | 8 | 9 | 2 | 8 | 4 | 2 |
| MF | GO:0016491 | oxidoreductase activity | 14 | 54 | 9 | 25 | 17 | 18 |

^*^BP: biological process, CC: cellular component, MF: molecular function
