# Supplementary material for: The Mechanism of Starch Over-Accumulation in Chlamydomonas reinhardtii High-Starch Mutants Identified by Comparative Transcriptome Analysis
Source: Front Microbiol. 2017 May 23;8:858. doi: 10.3389/fmicb.2017.00858 (PMC5440458; doi:10.3389/fmicb.2017.00858)
Supplement: Supplementary file 5 [file Table_5.DOCX]

**Supplemental Table 5**. Information for genes involved in starch biosynthesis

| **Gene locus** | **KEGG description** | **EC number** |
| --- | --- | --- |
| Cre16.g683450.t1.2 | ADP-glucose pyrophosphorylase large subunit; K00975 glucose-1-phosphate adenylyltransferase | 2.7.7.27 |
| Cre03.g194700.t1.1 | AGL1; alpha glucosidase | 3.2.1.20 |
| Cre08.g384750.t1.1 | alpha-amylase-like protein; K01176 alpha-amylase | 3.2.1.1 |
| Cre08.g385500.t1.2 | AMYA1; alpha-amylase | 3.2.1.1 |
| Cre08.g362450.t1.2 | AMYA2; alpha-amylase | 3.2.1.1 |
| Cre06.g307150.t1.1 | AMYB1; beta-amylase | 3.2.1.2 |
| Cre06.g270350.t1.1 | AMYB2; beta-amylase | 3.2.1.2 |
| Cre01.g044100.t1.2 | AMYB3; beta-amylase | 3.2.1.2 |
| Cre02.g095126.t1.1 | DPE1; 4-alpha-glucanotransferase | 2.4.1.25 |
| Cre03.g185350.t1.1 | FAP228; flagellar associated protein, callose synthase-like protein | 2.4.1.34 |
| Cre03.g169400.t1.2 | GAD1; UDP-D-glucuronic acid decarboxylase | 4.1.1.35 |
| Cre06.g278210.t1.1 | GPM1a; phosphoglucomutase | 5.4.2.2 |
| Cre01.g012600.t1.1 | GPM2; phosphoglucomutase | 5.4.2.2 |
| Cre03.g198200.t1.1 | GTR11; glycosyl transferase | 2.4.1.34 |
| Cre04.g214650.t1.1 | GTR12; glycosyl transferase | 2.4.1.34 |
| Cre06.g302050.t1.2 | GTR12; glycosyl transferase | 2.4.1.34 |
| Cre12.g524000.t1.2 | GTR7; glycosyl transferase | 2.4.1.14 |
| Cre02.g117500.t1.2 | HXK1; hexokinase | 2.7.1.1 |
| Cre12.g488000.t1.2 | hypothetical protein; K01193 beta-fructofuranosidase | 3.2.1.26 |
| Cre03.g175400.t1.2 | PGI1; phosphoglucose isomerase | 5.3.1.9 |
| Cre07.g336950.t1.1 | PHOA; starch phosphorylase | 2.4.1.1 |
| Cre12.g552200.t1.2 | PHOB; starch phosphorylase | 2.4.1.1 |
| Cre06.g289850.t1.1 | SBE1; starch branching enzyme | 2.4.1.18 |
| Cre06.g270100.t1.1 | SBE2; starch branching enzyme | 2.4.1.18 |
| Cre10.g444700.t1.1 | SBE3; starch branching enzyme | 2.4.1.18 |
| Cre06.g278143.t1.1 | SNE4; NAD-dependent epimerase/dehydratase | 5.1.3.6 |
| Cre12.g521700.t1.2 | soluble starch synthase; K00703 starch synthase | 2.4.1.21 |
| Cre04.g215150.t1.2 | SSS1; soluble starch synthase | 2.4.1.21 |
| Cre03.g185250.t1.2 | SSS2; soluble starch synthase II | 2.4.1.21 |
| Cre13.g579582.t1.1 | SSS3; soluble starch synthase, starch synthase III | 2.4.1.21 |
| Cre13.g579598.t1.1 | SSS3; soluble starch synthase, starch synthase III | 2.4.1.21 |
| Cre16.g665800.t1.2 | SSS4; soluble starch synthase | 2.4.1.21 |
| Cre13.g567950.t1.2 | STA1; ADP-glucose pyrophosphorylase large subunit | 2.7.7.27 |
| Cre03.g181500.t1.2 | STA11; 4-alpha-glucanotransferase | 2.4.1.25 |
| Cre06.g282000.t1.1 | STA3; soluble starch synthase III | 2.4.1.21 |
| Cre03.g188250.t1.2 | STA6; ADP-glucose pyrophosphorylase small subunit | 2.7.7.27 |
| Cre06.g278221.t1.1 | TPS1; trehalose-6-phosphate synthase/phosphatase | 3.1.3.12, 2.4.1.15 |
| Cre12.g497750.t1.2 | TPS2; trehalose 6-phosphate phosphatase | 3.1.3.12 |
| Cre16.g686200.t1.1 | TPS3; trehalose-6-phosphate synthase/phosphatase | 3.1.3.12, 2.4.1.15 |
| Cre16.g662350.t1.1 | TPS3; trehalose-6-phosphate synthase/phosphatase | 3.1.3.12, 2.4.1.15 |
| Cre03.g195600.t1.2 | trehalase-like protein; K01194 alpha,alpha-trehalase | 3.2.1.28 |
| Cre12.g532450.t1.2 | UDP-glucose 6-dehydrogenase; K00012 UDPglucose 6-dehydrogenase | 1.1.1.22 |
| Cre07.g357200.t1.2 | UGD1; UDP-glucose dehydrogenase | 1.1.1.22 |
| Cre06.g278185.t1.1 | UGD2; UDP-glucose dehydrogenase | 1.1.1.22 |
| Cre04.g229700.t1.2 | UGP1; UDP-glucose pyrophosphorylase | 2.7.7.9 |
