# Supplementary material for: The Mechanism of Starch Over-Accumulation in Chlamydomonas reinhardtii High-Starch Mutants Identified by Comparative Transcriptome Analysis
Source: Front Microbiol. 2017 May 23;8:858. doi: 10.3389/fmicb.2017.00858 (PMC5440458; doi:10.3389/fmicb.2017.00858)
Supplement: Supplementary file 6 [file Table_6.DOCX]

**Supplemental Table 6**. Information for genes involved in glycolysis

| **Gene locus** | **KEGG description** | **EC number** |
| --- | --- | --- |
| Cre01.g071662.t1.1 | ACS1; acetyl CoA synthetase | [EC:6.2.1.1] |
| Cre01.g055408.t1.1 | ACS2; acetyl CoA synthetase | [EC:6.2.1.1] |
| Cre07.g353450.t1.2 | ACS3; acetyl CoA synthetase | [EC:6.2.1.1] |
| Cre06.g262050.t1.2 | AEP1; aldose-1-epimerase | [EC:5.1.3.3] |
| Cre12.g500150.t1.1 | aldehyde dehydrogenase; K00128 aldehyde dehydrogenase (NAD+) | [EC:1.2.1.3] |
| Cre09.g386735.t1.1 | DLA1; dihydrolipoamide acetyltransferase | [EC:2.3.1.12] |
| Cre03.g158900.t1.2 | DLA2; dihydrolipoamide acetyltransferase | [EC:2.3.1.12] |
| Cre06.g252550.t1.1 | DLA3; dihydrolipoamide acetyltransferase | [EC:2.3.1.12] |
| Cre01.g016514.t1.1 | DLD2; dihydrolipoamide dehydrogenase | [EC:1.8.1.4] |
| Cre01.g006950.t1.1 | FBA1; fructose-1,6-bisphosphate aldolase | [EC:4.1.2.13] |
| Cre02.g093450.t1.2 | FBA2; fructose-1,6-bisphosphate aldolase | [EC:4.1.2.13] |
| Cre05.g234550.t1.2 | FBA3; fructose-1,6-bisphosphate aldolase | [EC:4.1.2.13] |
| Cre02.g115650.t1.2 | FBA4; fructose-1,6-bisphosphate aldolase | [EC:4.1.2.13] |
| Cre12.g510650.t1.2 | FBP1; fructose-1,6-bisphosphatase | [EC:3.1.3.11] |
| Cre12.g543400.t1.2 | FDH1; formaldehyde dehydrogenase | [EC:1.1.1.1, EC:1.1.1.284] |
| Cre12.g485150.t1.2 | GAP1a; glyceraldehyde 3-phosphate dehydrogenase, dominant splicing variant | [EC:1.2.1.12] |
| Cre07.g354200.t1.2 | GAP2; glyceraldehyde 3-phosphate dehydrogenase | [EC:1.2.1.12] |
| Cre12.g556600.t1.2 | GAPN1; glyceraldehyde 3-phosphate dehydrogenase, nonphosphorylating | [EC:1.2.1.9] |
| Cre18.g749847.t1.1 | GCSL; dihydrolipoyl dehydrogenase | [EC:1.8.1.4] |
| Cre06.g278210.t1.1 | GPM1a; phosphoglucomutase | [EC:5.4.2.2] |
| Cre01.g012600.t1.1 | GPM2; phosphoglucomutase | [EC:5.4.2.2] |
| Cre02.g117500.t1.2 | HXK1; hexokinase | [EC:2.7.1.1] |
| Cre16.g677450.t1.2 | hypothetical protein; K01792 glucose-6-phosphate 1-epimerase | [EC:5.1.3.15] |
| Cre07.g347100.t1.2 | hypothetical protein; K01792 glucose-6-phosphate 1-epimerase | [EC:5.1.3.15] |
| Cre13.g568600.t1.1 | hypothetical protein; K03103 multiple inositol-polyphosphate phosphatase / 2,3-bisphosphoglycerate 3-phosphatase | [EC:3.1.3.80, EC:3.1.3.62] |
| Cre02.g141400.t1.2 | PCK1b; phosphoenolpyruvate carboxykinase, splice variant [EC:4.1.1.49] | [EC:4.1.1.49] |
| Cre07.g337650.t1.2 | PDC1; mitochondrial pyruvate dehydrogenase complex, E1 component, alpha subunit | [EC:1.2.4.1] |
| Cre03.g165700.t1.1 | PDC3; mitochondrial pyruvate dehydrogenase complex, E1 component, alpha subunit | [EC:4.1.1.1] |
| Cre16.g677026.t1.1 | PDH1a; pyruvate dehydrogenase E1 beta subunit | [EC:1.2.4.1] |
| Cre03.g194200.t1.2 | PDH2; pyruvate dehydrogenase E1 beta subunit | [EC:1.2.4.1] |
| Cre06.g262900.t1.2 | PFK1; phosphofructokinase family protein | [EC:2.7.1.11] |
| Cre12.g553250.t1.2 | PFK2; phosphofructokinase family protein | [EC:2.7.1.11] |
| Cre11.g467557.t1.1 | PFK3; phosphofructokinase | [EC:2.7.1.11] |
| Cre11.g467552.t1.1 | PFK3; phosphofructokinase | [EC:2.7.1.11] |
| Cre12.g513200.t1.2 | PGH1; enolase | [EC:4.2.1.11] |
| Cre03.g175400.t1.2 | PGI1; phosphoglucose isomerase | [EC:5.3.1.9] |
| Cre11.g467770.t1.1 | PGK1; phosphoglycerate kinase | [EC:2.7.2.3] |
| Cre06.g272050.t1.2 | PGM1a; phosphoglycerate mutase | [EC:5.4.2.12] |
| Cre10.g460300.t1.2 | PGM2; phosphoglycerate mutase | [EC:5.4.2.12] |
| Cre05.g232550.t1.2 | PGM4; phosphoglycerate mutase | [EC:5.4.2.12] |
| Cre03.g166950.t1.2 | PGM5; phosphoglycerate mutase | [EC:5.4.2.11] |
| Cre12.g533550.t1.1 | PYK1; pyruvate kinase | [EC:2.7.1.40] |
| Cre06.g280950.t1.2 | PYK2; pyruvate kinase | [EC:2.7.1.40] |
| Cre05.g234700.t1.1 | PYK3a; pyruvate kinase | [EC:2.7.1.40] |
| Cre03.g144847.t1.1 | PYK4a; pyruvate kinase | [EC:2.7.1.40] |
| Cre02.g147900.t1.1 | PYK5; pyruvate kinase | [EC:2.7.1.40] |
| Cre01.g029300.t1.2 | TPIC; triose phosphate isomerase | [EC:5.3.1.1] |
